# Children with access to improved sanitation but not improved water are at lower risk of stunting compared to children without access: a cohort study in Ethiopia, India, Peru, and Vietnam

**DOI:** 10.1186/s12889-017-4033-1

**Published:** 2017-01-23

**Authors:** Kirk A. Dearden, Whitney Schott, Benjamin T. Crookston, Debbie L. Humphries, Mary E. Penny, Jere R. Behrman, The Young Lives Determinants and Consequences of Child Growth Project Team, Santiago Cueto, Le Thuc Duc, Javier Escobal, Lia Fernald, Shaik Galab, Priscila Hermida, Andreas Georgiadis, Elizabeth Lundeen, Subha Mani, Aryeh Stein, Tassew Woldehanna

**Affiliations:** 1 IMA World Health, Dar es Salaam, Tanzania; 2 Population Studies Center, University of Pennsylvania, Philadelphia, PA USA; 3 Department of Health Science, Brigham Young University, Provo, UT USA; 4 Department of Epidemiology, Yale University, New Haven, CT USA; 5 Instituto de Investigación Nutricional, Lima 12, Peru

**Keywords:** Water, Sanitation, Stunting, Thinness

## Abstract

**Background:**

This study’s purpose was to understand associations between water, sanitation, and child growth.

**Methods:**

We estimated stunting (height-for-age Z score <−2 SD) and thinness (BMI-Z <−2 SD) risk ratios using data from 7,715 Ethiopian, Indian, Peruvian, and Vietnamese children from the Young Lives study.

**Results:**

In unadjusted models, household access to improved water and toilets was often associated with reduced stunting risk. After adjusting for child, household, parent, and community variables, access to improved water was usually not associated with stunting nor thinness except in Ethiopia where access to improved water was associated with reduced stunting and thinness at 1y and 5y. In contrast, in both unadjusted and adjusted models, stunting at 1y was less common among children with good toilet access than among those without access and this difference persisted when children were 5y and 8y. For example, in adjusted estimates, Vietnamese 5y olds with access to improved toilets had relative stunting risk at 8y 0.62-0.68 that of 5y olds with no access to improved toilets. Water and toilets were rarely associated with thinness.

**Conclusions:**

Results from our study indicate that access to improved sanitation is more frequently associated with reduced stunting risk than access to improved water. However, additional studies are needed before drawing definitive conclusions about the impact of toilets relative to water. This study is the first to our knowledge to demonstrate the robust and persistent importance of access to improved toilets in infancy, not only during the first year but continuing into childhood. Additional longitudinal investigations are needed to determine concurrent and long-term associations of WASH with stunting and thinness.

**Electronic supplementary material:**

The online version of this article (doi:10.1186/s12889-017-4033-1) contains supplementary material, which is available to authorized users.

## Background

Suboptimum early childhood growth is associated with lifelong negative outcomes [1–5]. Diet and infection impacts on nutritional status are well-known [6, 7]. Evidence indicates that poor water, sanitation^1^, and hygiene (WASH) increase risk of infections, and infections influence growth. For example, improved sanitation is associated with reduced diarrhea [8–10] and helminth infections [10–13]. Two studies document that human excreta disposal and sewerage reduced diarrhea [14, 15]. Another recent study [16] found that Indian latrine promotion and construction did not reduce open defecation much and that reductions in diarrhea among children under 5y were negligible. Inadequate handwashing increases risks of soil-transmitted helminths [10, 17], diarrhea [8, 18–21], and pneumonia [18, 20]. Diarrhea is associated with stunting [22], and children with helminth infection gained significant weight and height [23] when treated. Mechanisms linking WASH and growth likely include diarrheal morbidity, parasitic infections, and environmental enteropathy (EE) [7, 24]. EE may be far more common than overt diarrheal illness [25], especially among people living in unhygienic conditions [26].

There are only a few rigorous studies that explore direct associations between WASH and growth [9, 16, 27–30]. Previous observational studies on access to improved water and growth find significant protective associations [28, 31], though some of these associations are not significant after covariate adjustments [29]. According to recent analysis of Demographic and Health Surveys (DHS) [30], in South Asia and Africa, access to improved water is one of the factors most strongly associated with stunting reductions between 1970 and 2015. However, other observational studies have found little or no significant protective associations between access to improved water and anthropometric outcomes [16, 29].

Several observational studies suggest that access to improved sanitation is associated with reduced stunting, including three analyses utilizing multi-country DHS data [9, 30, 32] and other studies in Bangladesh [33], India [34] and Brazil [35]. A recent decomposition analysis of improvement in HAZ from 2005 to 2010 among children under age 5y in Cambodia concluded that reductions in exposure to open defecation were associated with most of the change in HAZ [36]. One study reported that in India open defecation was associated with greater stunting prevalence [37] and recent analysis of DHS surveys [30] identified sanitation access as one of three covariates with the greatest potential to reduce stunting.

Intervention studies have failed to find WASH impacts on anthropometry, and one publication [38] suggests that programs that increase access to improved water had no effect on stunting. Two non-randomized studies [39, 40] and one randomized study [41] showed no impact of interventions addressing water, sanitation, and hygiene-either singly or in combination-on height-for-age z-scores (HAZ) or weight-for-age z-scores (WAZ). An Indian cluster-randomized trial [16] found no impact of latrine promotion and construction on HAZ or WAZ, a finding also reported elsewhere [42]. A meta-analysis [43] of five cluster-randomized trials showed no effect of interventions to improve water access and handwashing on underweight and only borderline statistically significant impacts on linear growth (or HAZ). However, results from a few randomized studies [44, 45], including an analysis of cluster randomized interventions in India, Indonesia, Mali and Tanzania [46], and another in Ethiopia [38], found positive improved sanitation impacts on mean HAZ. The analysis by Gertler et al. [46] notes the importance of community context, concluding that impact on HAZ on improvements in individual and household sanitation behavior is limited as long as the community context does not also improve. Interventions included efforts to protect water supplies and sanitation education regarding soap use, handwashing practices, sanitary facility construction, household cleanliness, separate animal housing, and keeping water clean.

This study’s purpose is to better understand associations between water, sanitation, and child growth^2^. Interview data on household, parent, and community measures, including household indicators of access to improved water and sanitation, were analyzed from three data rounds from a four-country cohort study (Ethiopia, India, Peru, Vietnam) with harmonized instruments and children in the same birth cohorts. We focus on stunting (HAZ < −2) and thinness (BMI-Z < −2) because we are interested in children in the left tails of the distributions. Our hypotheses are:

1. Access to improved drinking water (“water”) when children are 1y, 5y, and 8y is associated with reduced risks of stunting and thinness, concurrently (1A) and subsequently (1B).
2. Access to improved toilet facilities (“toilets”) at these ages is associated with reduced risks of stunting and thinness, concurrently (2A) and subsequently (2B).
3. Associations between access to improved drinking water and reductions in risks of stunting and thinness remain after adjusting for child, household, parent, and community covariates. Similarly, associations between access to improved toilet facilities and reduced risks of stunting and thinness remain after adjusting for these same covariates.
4. Associations between inadequate access to water and toilets and stunting and thinness are likely stronger in infancy than at older ages.

Our hypotheses were pre-specified and not the result of preliminary explorations.

**Fig. 1:**
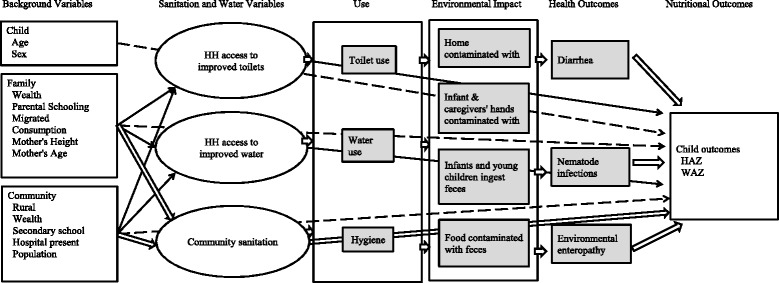
Conceptual framework for Water, Sanitation, and Hygiene (WASH) and child growth. Analysis is of the relationship specified by solid and dashed arrows, not wide arrows. Variables in shaded boxes are not available for analysis

Figure 1 illustrates our conceptual framework. We focus on household water and sanitation.

## Methods

### Study design and participants

We use Young Lives (YL) Younger Cohort data on 8062 children in Ethiopia, India, Peru, and Vietnam (http://www.younglives.org.uk). The Young Lives project is a study of children growing up in resource-poor settings during a time of renewed focus on economic development and corresponds with the implementation of the Millennium Development Goals (MDGs). Children were enrolled in 2002 at 6–17 months (“1y”), and followed in 2006–2007 at 4–5y (“5y”), and 2009–2010 at 7–8y (“8y”). The sampling strategy was described previously [47]. Multistage sampling design was pro-poor, with the first stage consisting of selection of 20 clusters that included food-insecure and varied areas of the country. In the case of Peru, the richest 5% of districts were excluded from the sample. While poor clusters were moderately oversampled, the final samples provided diverse representation of social, geographic, and demographic groups. The sample in India was from Andhra Pradesh and Telangana, while all three remaining countries used nationwide samples. Within each cluster ~100 households with children 6–17.9 months were randomly selected. Due to extensive efforts made by YL researchers and staff to closely track study participants, even among those who migrated, attrition for the study was low over the first three rounds, ranging from 2.2% in Vietnam to 5.7% in Ethiopia for an overall study loss of 4.4%. Attrition occurred largely as a result of mobility, though mortality accounted for a loss of 1.7% of participants [47]. For this analysis we excluded children who were not the target age (6–17.9 months) at recruitment, whose absolute value of change in HAZ between rounds exceeded four, or whose absolute value of HAZ exceeded five in any round. In all analyses, we used multiple imputation for missing values [48] of dependent variables and covariates employing the ice command in Stata 12.1 and 25 imputations. Our final sample, after accounting for exclusion criteria, attrition, and multiple imputation, was 7715 children (Ethiopia: 1892 (94.8% of initial observations), India: 1919 (95.5%), Peru: 1999 (97.4%), and Vietnam: 1905 (95.4%)).

### Study indicators

#### Anthropometry

Length (at 1y) and height (at 5y and 8y) were measured to 1 mm using standardized length boards and stadiometers. Weight was measured using platform scales or clock balances. HAZ and BMI-Z were calculated using WHO 2006 standards for children 0–59 months [49] and WHO 2007 standards for children 5–8y [50]. As is conventional, stunting was defined as HAZ < −2 and thinness as BMI-Z < −2.

#### Access to improved water and sanitation

Measures of households’ main source of water and sanitation were available for each age (1y, 5y, 8y) with access to “improved” water classified as borehole, tube well, piped water, public standpipe, community water tank, rain water, and other protected water source; and access to “improved” toilets classified as household flush toilet, piped sewer system connection, septic system connection, composting toilet, and pit latrine.

#### Other variables

Household variables included child’s sex and age; asset index in concurrent round^3^; mother’s height, age, and completed schooling grades; father’s completed schooling grades; household migration between rounds; and urban/rural residence at 1y. Community characteristics at 1y included hospital presence, secondary school presence, population^4^, and community wealth^5^. Additional file 1: Table S1 (available online as a supplementary table) details all measures used for this study, including information about when each was collected.

### Statistical analyses

To estimate risk ratios, by country, we conducted modified Poisson regressions [51] for stunting and thinness at 1y, 5y, and 8y, regressing first on household improved water and sanitation indicators alone, and then adjusting for child characteristics, then including household, parent, and community characteristics in separate models. To further examine relationships, we conducted multivariate OLS regressions of HAZ and BMI-Z at 1y, 5y, and 8y, first on water and sanitation indicators alone, and then adjusting for the child, household, parent, and community characteristics, and allowing for community clustered errors^6^. We also estimated pooled models with country interactions to test for heterogeneous associations across countries. We present results by country to account for differences across geographic regions and cultures. In the Discussion, we review findings as a whole, relative to our hypotheses.

## Results

### Stunting and thinness (Table 1)

**Table 1:** Descriptive statistics

	Ethiopia (*n* = 1,892)			India (*n* = 1,919)			Peru (*n* = 1,999)			Vietnam (*n* = 1,905)		
	Mean/%	SD	No. Missing^a^	Mean/%	SD	No. Missing^a^	Mean/%	SD	No. Missing^a^	Mean/%	SD	No. Missing^a^
Stunting and low BMI												
Stunted (HAZ < −2) at age ~1y	40.1		48	29.9		17	27.6		10	19.7		3
Stunted (HAZ < −2) at age ~5y	30.7		75	34.7		63	32.5		93	24.3		39
Stunted (HAZ < −2) at age ~8y	20.1		98	28.4		78	19.9		113	19.0		51
Low BMI (zBMI < −2) at age ~1y	16.1		137	18.9		18	1.9		11	3.8		3
Low BMI (zBMI < −2) at age ~5y	8.5		75	18.9		63	0.4		93	3.9		39
Low BMI (zBMI < −2) at age ~8y	21.6		98	27.4		78	0.9		114	11.7		60
Household level water and toilets												
Improved water at age ~1y	53.9		0	75.5		0	80.7		0	10.2		0
Improved water at age ~5y	82.4		70	95.0		57	88.0		86	83.6		29
Improved water at age ~8y	89.0		95	96.9		76	92.0		105	87.5		0
Improved toilets at age ~1y	21.3		0	26.5		0	77.6		0	49.2		0
Improved toilets at age ~5y	41.1		70	32.9		57	85.3		86	55.0		29
Improved toilets at age ~8y	57.0		95	35.1		76	91.1		105	60.6		0
Child characteristics												
Age in months at age ~1y	11.6	3.6	0	11.7	3.4	0	11.5	3.5	0	11.7	3.1	0
Age in months at age ~5y	61.8	3.8	72	64.2	3.8	57	63.5	4.7	86	63.0	3.5	29
Age in months at age ~8y	96.9	3.9	97	95.4	3.9	77	94.9	3.6	105	96.5	3.5	47
Child is female	47.5		0	46.4		0	50.2		0	48.7		0
Parental characteristics												
Mother’s height	158.6	6.1	223	151.6	6.0	93	150.0	5.5	182	152.2	5.9	51
Mother’s completed schooling (grades attained)	3.0	3.9	89	3.7	4.4	61	7.8	4.4	101	7.0	4.0	44
Father’s completed schooling (grades attained)	4.9	4.3	154	5.6	5.0	60	9.1	3.9	145	7.7	4.0	77
Mother’s age	27.4	6.3	51	23.6	4.3	9	26.8	6.8	15	27.1	5.7	5
Household characteristics												
Household size at age ~1y	5.7	2.2	0	5.4	2.4	0	5.7	2.3	0	4.9	1.8	0
Household size at age ~5y	6.0	2.1	72	5.5	2.2	57	5.5	2.1	86	4.6	1.5	29
Household size at age ~8y	6.2	2.0	95	5.4	2.3	76	5.4	1.9	105	4.6	1.5	29
Changed communities between ages ~1y and ~5y	14.0		72	9.8		57	47.7		86	15.6		29
Changed communities between ages ~5y and ~8y	9.7		97	2.5		77	77.4		131	0.2		46
Asset index at age ~1y	0.0	1.9	1	0.0	1.9	0	0.0	2.0	0	0.0	2.0	0
Asset index at age ~5y	0.0	2.1	71	0.0	2.0	57	0.0	2.1	86	0.0	1.9	29
Asset index at age ~8y	0.0	2.1	95	0.0	1.9	76	0.0	2.0	105	0.0	2.0	0
Urban residence	35.7		0	26.0		0	66.5		0	19.9		0
Other community characteristics												
Community wealth	0.0	2.6	96	0.0	2.0	78	0.0	2.5	132	0.0	2.2	29
Community has hospital	32.6		96	47.9		44	35.2		0	90.0		0
Community has secondary school	36.9		96	44.9		44	77.9		0	98.3		0
Community population	8,149.2	7,265.0	96	3,103.9	3,659.0	149	4,255.4	3,923.9	207	10,376.1	5,834.3	0

^a^This column reflects the number of missing (and thus, imputed with multiple imputation) observations per variable

Percentages of children stunted at 1y ranged from 19.7% (Vietnam) to 40.1% (Ethiopia). At 8y, percentages remained similar to those at 1y in Indian and Vietnamese study populations, but declined in Ethiopian and Peruvian study populations. Percentages of thinness at 1y were lowest in the Peruvian sample (1.9%) and highest in the Indian sample (18.9%), with increases between 1y and 8y in the Indian (18.9 to 27.4%) and Vietnamese samples (3.8 to 11.6%).

### Household access to improved water and sanitation (Table 1)

Vietnamese households had the least access to improved water for their main source when children were 1y (10.2%), and the largest increase in access by 8y (to 87.5%). By 8y, all four country study samples had almost universal household access to improved water (87.5 to 96.9%), which implies limited explanatory power in the estimates below. Ethiopian (21.3%) and Indian (26.5%) study samples had lowest access to improved toilets at 1y. Access increased in Ethiopia (57.0%) and India (35.1%) by 8y.

### Ethiopia

#### Hypothesis 1 (Water, stunting, and thinness, unadjusted)

Stunting at 1y was less common among children with access to improved water (Table 2, column I: 0.84, 95% CI: 0.75–0.94) and this difference persisted when the children were 5y and 8y. Children with access to improved water were no more likely to be stunted concurrently, either at 5y or at 8y. Access to improved water at 1y and again at 5y (but not 8y) was associated with concurrent thinness (Table 2, column I). Access to improved water was not associated with subsequent thinness.

**Table 2:** Ethiopia: Modified Poisson regression models for stunting [HAZ < −2] and thinness [BMI < −2] on improved water and sanitation facilities, relative risk ratios and confidence intervals

	Unadjusted	Unadjusted	Child Adjusted	Child and Household Adjusted	Child, Household, and Parent Adjusted	Child, Household, Parent, and Community Adjusted
*n* = 1,892	I	II	III	IV	V	VI
Stunting at age ~1y						
Improved water at age ~1y	0.84**		0.91	1.04	1.02	1.02
	[0.75–0.94]		[0.81–1.01]	[0.92–1.16]	[0.91–1.15]	[0.90–1.15]
Improved toilets at age ~1y		0.60**	0.62**	0.68**	0.74**	0.78**
		[0.50–0.71]	[0.52–0.74]	[0.57–0.81]	[0.62–0.88]	[0.64–0.93]
Stunting at age ~5y						
Improved water at age ~1y	0.75**		0.83**	0.97	0.96	0.95
	[0.65–0.87]		[0.72–0.95]	[0.84–1.13]	[0.83–1.11]	[0.81–1.10]
Improved water at age ~5y	0.97		1.02	1.11	1.12	1.07
	[0.81–1.16]		[0.85–1.21]	[0.93–1.32]	[0.94–1.32]	[0.90–1.28]
Improved toilets at age ~1y		0.46**	0.49**	0.56**	0.61**	0.56**
		[0.36–0.59]	[0.38–0.62]	[0.44–0.71]	[0.48–0.77]	[0.43–0.71]
Improved toilets at age ~5y		0.95	0.95	0.94	0.98	0.98
		[0.82–1.10]	[0.82–1.10]	[0.81–1.09]	[0.85–1.13]	[0.84–1.13]
Stunting at age ~8y						
Improved water at age ~1y	0.74**		0.83	1	0.99	0.97
	[0.61–0.89]		[0.68–1.01]	[0.82–1.22]	[0.81–1.21]	[0.79–1.20]
Improved water at age ~5y	0.83		0.85	0.93	0.93	0.92
	[0.66–1.06]		[0.67–1.08]	[0.74–1.17]	[0.73–1.18]	[0.72–1.16]
Improved water at age ~8y	0.83		0.82	0.91	0.91	0.91
	[0.63–1.10]		[0.63–1.08]	[0.69–1.20]	[0.69–1.20]	[0.69–1.20]
Improved toilets at age ~1y		0.49**	0.54**	0.64**	0.68**	0.64**
		[0.36–0.67]	[0.40–0.74]	[0.48–0.87]	[0.50–0.92]	[0.47–0.87]
Improved toilets at age ~5y		1.01	1.04	1.07	1.1	1.13
		[0.82–1.23]	[0.85–1.27]	[0.88–1.30]	[0.91–1.33]	[0.93–1.38]
Improved toilets at age ~8y		1.16	1.11	0.99	1.02	1.02
		[0.96–1.41]	[0.91–1.35]	[0.81–1.21]	[0.84–1.25]	[0.83–1.27]
Thinness at age ~1y						
Improved water at age ~1y	0.68**		0.72**	1.02	1	1.02
	[0.55–0.85]		[0.58–0.90]	[0.81–1.28]	[0.79–1.25]	[0.81–1.29]
Improved toilets at age ~1y		0.67**	0.74*	0.94	0.99	0.92
		[0.50–0.90]	[0.54–0.99]	[0.69–1.27]	[0.73–1.34]	[0.67–1.24]
Thinness at age ~5y						
Improved water at age ~1y	1.1		1.02	1.08	1.07	1.07
	[0.80–1.51]		[0.74–1.42]	[0.77–1.51]	[0.76–1.51]	[0.74–1.53]
Improved water at age ~5y	0.55**		0.55**	0.56**	0.57**	0.57**
	[0.39–0.79]		[0.38–0.78]	[0.39–0.80]	[0.39–0.81]	[0.39–0.82]
Improved toilets at age ~1y		1.33	1.42	1.46*	1.46*	1.56**
		[0.94–1.90]	[0.99–2.04]	[1.01–2.12]	[1.00–2.14]	[1.05–2.32]
Improved toilets at age ~5y		0.91	0.98	0.97	0.96	1
		[0.66–1.26]	[0.71–1.35]	[0.70–1.35]	[0.69–1.34]	[0.70–1.41]
Thinness at age ~8y						
Improved water at age ~1y	0.9		0.9	1.02	1.03	1
	[0.75–1.09]		[0.74–1.10]	[0.84–1.25]	[0.84–1.26]	[0.82–1.23]
Improved water at age ~5y	0.87		0.89	0.94	0.94	0.94
	[0.68–1.10]		[0.70–1.12]	[0.75–1.19]	[0.75–1.19]	[0.74–1.20]
Improved water at age ~8y	0.83		0.85	0.91	0.93	0.94
	[0.63–1.10]		[0.64–1.12]	[0.69–1.21]	[0.70–1.22]	[0.71–1.25]
Improved toilets at age ~1y		0.94	0.99	1.09	1.13	1.2
		[0.75–1.19]	[0.78–1.26]	[0.86–1.39]	[0.89–1.44]	[0.94–1.54]
Improved toilets at age ~5y		0.85	0.88	0.9	0.92	0.96
		[0.70–1.03]	[0.72–1.07]	[0.75–1.10]	[0.76–1.12]	[0.79–1.18]
Improved toilets at age ~8y		0.97	0.94	0.89	0.9	0.95
		[0.80–1.16]	[0.78–1.14]	[0.73–1.08]	[0.74–1.09]	[0.78–1.17]

*p < 0 · 05, **p < 0 · 01. All adjusted models include both improved water and improved toilets. Child variables include age in months at outcome and child sex. Household variables are asset index, household size, and household moved between rounds when there is more than one round of data on household toilet and water. Parental variables are age of mother, height of mother, years of schooling of mother, and years of schooling of father. Community variables are urban residence, community population, community wealth, presence of a community hospital, and community has public secondary school

#### Hypothesis 2 (Toilets, stunting, and thinness, unadjusted)

Children with access to improved toilets at 1y had about half the risk of being stunted at 1y compared to children without access at 1y, and this difference persisted when the children were 5y and 8y (Table 2, column II). There were no significant associations between access to improved toilets at 5y and 8y and the risk of stunting at 5y and 8y. Toilet access was rarely associated with concurrent or subsequent thinness (Table 2, column II).

#### Hypothesis 3 (Adjustment for covariates)

Adjusting for child, household, parent, and community characteristics, there were no significant associations between access to improved water and risk of stunting (Table 2, columns III–VI) with the exception of access to improved water at 1y and subsequent lower risk of stunting at 5y. Access to improved water at 5y remained significantly associated with less thinness at 5y, even when adjusting for covariates (relative risk ratios from 0.55 to 0.57). Associations between toilet access at 1y and lower risk of stunting at 1y, 5y and 8y remained after adjusting for all covariates.

#### Hypothesis 4 (Infancy versus older ages)

Inadequate toilet access in infancy was no more likely to be associated with risk of stunting and thinness than inadequate toilet access at older ages. For example, the risk ratio of improved toilets at 1y for stunting at 1y (0.78) was similar to the coefficient for toilets at 1y and stunting at 5y and 8y (0.56 and 0.64, respectively; Table 2, column VI). Other associations (water and stunting, water and thinness, and toilets and thinness) were largely not significant.

### India

#### Hypothesis 1 (Water, stunting, and thinness, unadjusted)

Stunting at 5y was less common among children with access to improved water at 5y than among those without access. This association persisted: access to improved water at 5y was associated with less risk of stunting at 8y (Table 3, column I). No other associations-either concurrent or subsequent-were observed. Access to improved water at 1y was associated with thinness at 1y, although unexpectedly, infants with access to improved water at 1y experienced 1.63 times (95% CI: 1.26, 2.11) the risk of thinness when compared with 1y olds without access to improved water (Table 3, column I).

**Table 3:** India: Modified Poisson regression models for stunting [HAZ < −2] and thinness [BMI < −2] on improved water and sanitation facilities, relative risk ratios and confidence intervals

	Unadjusted	Unadjusted	Child Adjusted	Child and Household Adjusted	Child, Household, and Parent Adjusted	Child, Household, Parent, and Community Adjusted
*n* = 1,919	I	II	III	IV	V	VI
Stunting at age ~1y						
Improved water at age ~1y	1.00		0.97	1.01	1.01	1.04
	[0.85–1.17]		[0.83–1.14]	[0.87–1.18]	[0.87–1.18]	[0.89–1.23]
Improved toilets at age ~1y		0.59**	0.58**	1	1.05	0.96
		[0.49–0.71]	[0.48–0.71]	[0.79–1.26]	[0.83–1.33]	[0.72–1.30]
Stunting at age ~5y						
Improved water at age ~1y	1.24**		1.22*	1.21*	1.22**	1.18**
	[1.06–1.45]		[1.04–1.42]	[1.04–1.41]	[1.05–1.42]	[1.01–1.39]
Improved water at age ~5y	0.70**		0.77*	0.84	0.87	0.84
	[0.55–0.87]		[0.61–0.96]	[0.67–1.05]	[0.70–1.08]	[0.68–1.05]
Improved toilets at age ~1y		0.56**	0.56**	0.68**	0.72**	0.76*
		[0.44–0.71]	[0.44–0.71]	[0.54–0.87]	[0.57–0.91]	[0.57–1.02]
Improved toilets at age ~5y		0.83	0.84	1.04	1.07	1.08
		[0.69–1.01]	[0.69–1.01]	[0.86–1.27]	[0.88–1.29]	[0.88–1.32]
Stunting at age ~8y						
Improved water at age ~1y	1.16		1.15	1.13	1.14	1.09
	[0.97–1.39]		[0.97–1.37]	[0.95–1.34]	[0.96–1.36]	[0.91–1.32]
Improved water at age ~5y	0.72*		0.82	0.89	0.92	0.93
	[0.53–0.96]		[0.62–1.09]	[0.68–1.18]	[0.70–1.20]	[0.71–1.23]
Improved water at age ~8y	0.86		0.91	1	0.99	0.98
	[0.59–1.27]		[0.63–1.32]	[0.69–1.44]	[0.69–1.42]	[0.68–1.40]
Improved toilets at age ~1y		0.60**	0.60**	0.66**	0.72**	0.83
		[0.44–0.81]	[0.44–0.82]	[0.48–0.90]	[0.52–0.98]	[0.59–1.16]
Improved toilets at age ~5y		0.87	0.88	0.98	1.02	1.05
		[0.67–1.13]	[0.68–1.14]	[0.76–1.27]	[0.79–1.31]	[0.81–1.37]
Improved toilets at age ~8y		0.69**	0.69**	0.83	0.84	0.88
		[0.54–0.88]	[0.54–0.87]	[0.64–1.07]	[0.65–1.09]	[0.67–1.14]
Thinness at age ~1y						
Improved water at age ~1y	1.63**		1.59**	1.60**	1.60**	1.42**
	[1.26–2.11]		[1.23–2.05]	[1.24–2.07]	[1.24–2.07]	[1.08–1.87]
Improved toilets at age ~1y		0.79*	0.78*	0.86	0.85	1.13
		[0.63–0.99]	[0.63–0.98]	[0.64–1.17]	[0.62–1.16]	[0.76–1.68]
Thinness at age ~5y						
Improved water at age ~1y	1.04		1.06	1.05	1.06	1.05
	[0.83–1.29]		[0.85–1.32]	[0.85–1.32]	[0.85–1.33]	[0.83–1.33]
Improved water at age ~5y	0.98		0.95	0.96	0.95	0.95
	[0.64–1.49]		[0.63–1.45]	[0.63–1.47]	[0.63–1.45]	[0.61–1.47]
Improved toilets at age ~1y		1.12	1.13	1.16	1.15	1.23
		[0.84–1.51]	[0.85–1.51]	[0.86–1.57]	[0.85–1.56]	[0.84–1.82]
Improved toilets at age ~5y		0.95	0.95	0.99	0.99	0.99
		[0.72–1.26]	[0.73–1.25]	[0.74–1.33]	[0.74–1.34]	[0.74–1.33]
Thinness at age ~8y						
Improved water at age ~1y	0.98		0.99	0.97	0.98	1.01
	[0.83–1.17]		[0.83–1.17]	[0.82–1.16]	[0.82–1.16]	[0.84–1.21]
Improved water at age ~5y	0.96		1.05	1.08	1.07	1.04
	[0.67–1.40]		[0.72–1.51]	[0.75–1.56]	[0.74–1.55]	[0.72–1.51]
Improved water at age ~8y	1.76		1.85	1.91	1.88*	1.85*
	[0.92–3.40]		[0.96–3.55]	[1.00–3.67]	[0.98–3.60]	[0.97–3.53]
Improved toilets at age ~1y		0.89	0.89	0.92	0.94	0.98
		[0.68–1.16]	[0.68–1.16]	[0.70–1.20]	[0.72–1.23]	[0.71–1.35]
Improved toilets at age ~5y		0.91	0.89	0.94	0.97	0.98
		[0.70–1.18]	[0.69–1.16]	[0.72–1.22]	[0.74–1.26]	[0.74–1.30]
Improved toilets at age ~8y		0.84	0.83	0.88	0.91	0.92
		[0.67–1.06]	[0.65–1.04]	[0.69–1.13]	[0.71–1.17]	[0.72–1.18]

*p < 0 · 05, **p < 0 · 01. All adjusted models include both improved water and improved toilets. Child variables include age in months at outcome and child sex. Household variables are asset index, household size, and household moved between rounds when there is more than one round of data on household toilet and water. Parental variables are age of mother, height of mother, years of schooling of mother, and years of schooling of father. Community variables are urban residence, community population, community wealth, presence of a community hospital, and community has public secondary school

#### Hypothesis 2 (Toilets, stunting, and thinness, unadjusted)

Children with access to improved toilets at 1y and 5y were at lower risk of concurrent stunting than children without such access. One-year-olds with access to improved toilets had lower stunting risk at subsequent ages (RR = 0.56–0.60; Table 3, column II) when compared to 1 year-olds with no toilet access. Improved toilets were only associated with reduced thinness at 1y (RR = 0.79, 95% CI: 0.63, 0.99).

#### Hypothesis 3 (Adjustment for covariates)

With covariate adjustments, associations between water and risk of stunting were not significant with the exception of access to improved water at 1y and less risk of stunting at 5y (Table 3, columns III–VI). Associations between access to improved toilets at 1y and less risk of stunting at 5y remained significant after adjusting for all covariates (Table 3, columns III–VI). Likewise, associations between access to improved toilets at 1y and less risk of stunting at 8y remained significant after adjusting for all but the community covariates (Table 3, columns III–V).

#### Hypothesis 4 (Infancy versus older ages)

After adjusting for all covariates, with the exception of the association between access to improved toilets at 1y and reduced risk of stunting at 5y, there were no significant associations at any age between water, toilets, stunting, and thinness (Table 3, column VI). Access to improved water at 1y and risk of stunting at 5y were associated but in this case, access to improved water was associated with an increased risk of stunting. Thus, inadequate access to improved water and toilets in infancy was more likely associated with less risk of stunting but not less risk of thinness compared to inadequate access to water and toilets at older ages.

### Peru

#### Hypothesis 1 (Water, stunting, and thinness, unadjusted)

The risk of stunting at 1y in the Peruvian sample was lower among children with access to improved water (Table 4, column I) and this difference persisted when children were 5y and 8y. At both 5y and 8y, children with access to improved water were less likely than children without access to be stunted concurrently. There were no significant associations between access to improved water and thinness at any age.

**Table 4:** Peru: Modified Poisson regression models for stunting [HAZ < −2] and thinness [BMI < −2] on improved water and sanitation facilities, relative risk ratios and confidence intervals

	Unadjusted	Unadjusted	Child Adjusted	Child and Household Adjusted	Child, Household, and Parent Adjusted	Child, Household, Parent, and Community Adjusted
*n* = 1,999	I	II	III	IV	V	VI
Stunting at age ~1y						
Improved water at age ~1y	0.74**		0.88	1.1	1.08	1.06
	[0.63–0.87]		[0.74–1.04]	[0.94–1.29]	[0.93–1.26]	[0.90–1.24]
Improved toilets at age ~1y		0.62**	0.65**	0.88	0.91	0.93
		[0.53–0.72]	[0.56–0.76]	[0.76–1.02]	[0.79–1.06]	[0.80–1.07]
Stunting at age ~5y						
Improved water at age ~1y	0.80**		0.95	1.09	1.08	1.08
	[0.69–0.93]		[0.82–1.11]	[0.94–1.26]	[0.94–1.24]	[0.94–1.24]
Improved water at age ~5y	0.63**		0.74**	0.93	0.94	0.93
	[0.53–0.74]		[0.63–0.87]	[0.80–1.08]	[0.81–1.09]	[0.81–1.07]
Improved toilets at age ~1y		0.62**	0.71**	0.84*	0.90*	0.93
		[0.54–0.72]	[0.61–0.82]	[0.73–0.96]	[0.79–1.02]	[0.81–1.05]
Improved toilets at age ~5y		0.71**	0.77**	0.92	0.91	0.9
		[0.61–0.83]	[0.66–0.90]	[0.79–1.06]	[0.79–1.05]	[0.78–1.04]
Stunting at age ~8y						
Improved water at age ~1y	0.77*		0.91	1.03	1.06	1.08
	[0.62–0.96]		[0.72–1.15]	[0.83–1.29]	[0.86–1.30]	[0.87–1.33]
Improved water at age ~5y	0.77		0.85	1.02	1.07	1.09
	[0.59–1.01]		[0.65–1.12]	[0.79–1.32]	[0.84–1.37]	[0.85–1.40]
Improved water at age ~8y	0.65**		0.71*	1	0.88	0.87
	[0.49–0.86]		[0.53–0.94]	[0.76–1.32]	[0.68–1.16]	[0.66–1.14]
Improved toilets at age ~1y		0.63**	0.66**	0.84	0.91	0.93
		[0.51–0.77]	[0.52–0.82]	[0.68–1.03]	[0.75–1.12]	[0.76–1.15]
Improved toilets at age ~5y		0.68**	0.72**	0.89	0.9	0.88
		[0.54–0.86]	[0.57–0.92]	[0.72–1.11]	[0.72–1.12]	[0.71–1.09]
Improved toilets at age ~8y		0.83	0.91	0.93	0.89	0.88
		[0.64–1.09]	[0.70–1.19]	[0.71–1.21]	[0.69–1.14]	[0.69–1.13]
Thinness at age ~1y						
Improved water at age ~1y	0.67		0.75	0.92	0.93	0.88
	[0.33–1.37]		[0.33–1.68]	[0.42–2.02]	[0.42–2.05]	[0.39–1.99]
Improved toilets at age ~1y		0.62	0.75	0.92	0.93	0.88
		[0.32–1.23]	[0.33–1.68]	[0.42–2.02]	[0.42–2.05]	[0.39–1.99]
Thinness at age ~5y						
Improved water at age ~1y	1.82		1.81	1.88	1.94	1.83
	[0.15–21.96]		[0.11–29.12]	[0.12–29.55]	[0.12–32.45]	[0.12–28.60]
Improved water at age ~5y	0.74		0.55	0.66	0.71	0.7
	[0.06–8.85]		[0.02–12.29]	[0.03–13.81]	[0.03–14.89]	[0.04–13.53]
Improved toilets at age ~1y		1.14	0.97	1.08	1.02	0.91
		[0.10–12.64]	[0.11–8.29]	[0.13–9.26]	[0.12–8.55]	[0.13–6.14]
Improved toilets at age ~5y		494.61	681.99	881.43	854.18	1,079.33
		[0.00–6.15e + 09]	[0.00–1.19e + 10]	[0.00–2.49e + 10]	[0.00–1.81e + 10]	[0.00–3.54e + 10]
Thinness at age ~8y						
Improved water at age ~1y	0.87		1.02	1.05	0.99	0.99
	[0.26–2.93]		[0.26–3.99]	[0.27–4.08]	[0.25–3.92]	[0.18–5.43]
Improved water at age ~5y	0.58		0.6	0.61	0.59	0.56
	[0.16–2.07]		[0.18–1.98]	[0.18–2.10]	[0.18–1.95]	[0.13–2.53]
Improved water at age ~8y	1.82		1.8	1.91	2.02	2.09
	[0.24–13.87]		[0.23–14.22]	[0.24–15.41]	[0.24–17.04]	[0.15–28.56]
Improved toilets at age ~1y		0.67	0.67	0.69	0.63	0.52
		[0.22–2.06]	[0.19–2.37]	[0.18–2.64]	[0.16–2.55]	[0.09–2.94]
Improved toilets at age ~5y		0.78	0.86	0.89	0.89	0.86
		[0.21–2.88]	[0.25–2.94]	[0.26–3.00]	[0.26–3.12]	[0.19–3.94]
Improved toilets at age ~8y		1.45	1.31	1.32	1.38	1.56
		[0.22–9.72]	[0.19–8.95]	[0.20–8.94]	[0.21–9.29]	[0.15–16.12]

*p < 0 · 05, **p < 0 · 01. All adjusted models include both improved water and improved toilets. Child variables include age in months at outcome and child sex. Household variables are asset index, household size, and household moved between rounds when there is more than one round of data on household toilet and water. Parental variables are age of mother, height of mother, years of schooling of mother, and years of schooling of father. Community variables are urban residence, community population, community wealth, presence of a community hospital, and community has public secondary school

#### Hypothesis 2 (Toilets, stunting, and thinness, unadjusted)

At all ages, children with access to improved toilets at age 1y or 5y were about two-thirds as likely as children without improved toilets to be stunted (RR = 0.62–0.71; Table 3, column II). Access to improved toilets was never associated with thinness.

#### Hypothesis 3 (Adjustment for covariates)

After adjusting for covariates, associations between access to improved water and risk of stunting largely disappeared (Table 4, columns III–VI). Associations between access to improved toilets and less risk of stunting were attenuated by adjusting for covariates; however, all associations remained significant after adjusting for child variables and, in the case of toilets at 1y and stunting at 5y, after adjusting for household and parent characteristics as well.

#### Hypothesis 4 (Infancy versus older ages)

After adjusting for child, parent, household, and community covariates, there were no significant associations at any age between access to improved water and risk of stunting and thinness (Table 4, column VI). Likewise, there were no significant associations at any age between access to improved toilets and stunting and thinness.

### Vietnam

#### Hypothesis 1 (Water, stunting, and thinness, unadjusted)

At 1y, 5y, and 8y, children who had access to improved water were less likely to be stunted, both concurrently and at subsequent ages (Table 5, column I). The magnitude of these associations is notable. For example, infants who had access to improved water at 1y experienced about one-fifth the risk of stunting at any age, relative to children who did not have access to improved water at any age. Only access to improved water at 1y was associated with reduced thinness (at 8y).

**Table 5:** Vietnam: Modified Poisson regression models for stunting [HAZ < −2] and thinness [BMI < −2] on improved water and sanitation facilities, relative risk ratios and confidence intervals

	Unadjusted	Unadjusted	Child Adjusted	Child and Household Adjusted	Child, Household, and Parent Adjusted	Child, Household, Parent, and Community Adjusted
*n* = 1,905	I	II	III	IV	V	VI
Stunting at age ~1y						
Improved water at age ~1y	0.22**		0.26**	0.52	0.48**	0.57
	[0.11–0.41]		[0.14–0.50]	[0.26–1.01]	[0.25–0.93]	[0.27–1.20]
Improved toilets at age ~1y		0.61**	0.71**	1.06	1.12	1.12
		[0.50–0.74]	[0.59–0.86]	[0.86–1.31]	[0.90–1.40]	[0.90–1.39]
Stunting at age ~5y						
Improved water at age ~1y	0.20**		0.23**	0.30**	0.34**	0.54
	[0.10–0.38]		[0.12–0.44]	[0.15–0.58]	[0.18–0.65]	[0.25–1.17]
Improved water at age ~5y	0.76**		0.93	1.14	1.14	1.13
	[0.63–0.92]		[0.76–1.13]	[0.94–1.39]	[0.95–1.37]	[0.94–1.36]
Improved toilets at age ~1y		0.80*	0.93	1.03	1.17	1.19
		[0.65–0.99]	[0.75–1.14]	[0.84–1.27]	[0.94–1.45]	[0.96–1.47]
Improved toilets at age ~5y		0.66**	0.73**	0.94	0.99	1.04
		[0.54–0.82]	[0.60–0.90]	[0.75–1.17]	[0.79–1.23]	[0.83–1.30]
Stunting at age ~8y						
Improved water at age ~1y	0.21**		0.30**	0.31**	0.38**	0.59
	[0.10–0.44]		[0.14–0.65]	[0.14–0.66]	[0.18–0.80]	[0.25–1.42]
Improved water at age ~5y	0.77*		0.97	0.99	1	1.05
	[0.60–0.98]		[0.76–1.24]	[0.79–1.24]	[0.80–1.24]	[0.84–1.32]
Improved water at age ~8y	0.60**		0.71**	0.95	0.9	0.87
	[0.46–0.77]		[0.55–0.91]	[0.73–1.23]	[0.69–1.16]	[0.66–1.14]
Improved toilets at age ~1y		0.72*	0.81	0.81	0.91	0.91
		[0.55–0.95]	[0.62–1.05]	[0.63–1.05]	[0.70–1.18]	[0.70–1.18]
Improved toilets at age ~5y		0.58**	0.62**	0.65**	0.67**	0.68**
		[0.44–0.77]	[0.48–0.82]	[0.50–0.85]	[0.51–0.88]	[0.52–0.89]
Improved toilets at age ~8y		0.76*	0.83	1.05	1.13	1.17
		[0.60–0.97]	[0.65–1.06]	[0.82–1.36]	[0.87–1.47]	[0.91–1.51]
Thinness at age ~1y						
Improved water at age ~1y	0.78		1.26	0.98	0.99	1.48
	[0.34–1.78]		[0.50–3.14]	[0.36–2.63]	[0.38–2.55]	[0.43–5.05]
Improved toilets at age ~1y		0.47**	0.45**	0.40**	0.50**	0.55**
		[0.29–0.77]	[0.26–0.77]	[0.23–0.70]	[0.28–0.88]	[0.31–0.99]
Thinness at age ~5y						
Improved water at age ~1y	0.27		0.41	0.43	0.39	0.64
	[0.07–1.08]		[0.10–1.72]	[0.10–1.80]	[0.09–1.64]	[0.12–3.42]
Improved water at age ~5y	0.79		1.01	1.05	1.05	1.06
	[0.45–1.40]		[0.56–1.82]	[0.56–1.97]	[0.55–1.99]	[0.54–2.09]
Improved toilets at age ~1y		0.49*	0.54	0.55	0.55*	0.53**
		[0.26–0.93]	[0.29–1.01]	[0.29–1.04]	[0.29–1.05]	[0.28–0.98]
Improved toilets at age ~5y		0.77	0.81	0.84	0.82	0.77
		[0.43–1.39]	[0.46–1.43]	[0.46–1.55]	[0.44–1.50]	[0.42–1.43]
Thinness at age ~8y						
Improved water at age ~1y	0.21**		0.25**	0.25**	0.25**	0.28**
	[0.09–0.51]		[0.10–0.60]	[0.10–0.61]	[0.10–0.63]	[0.08–0.91]
Improved water at age ~5y	0.8		0.91	0.92	0.93	0.95
	[0.56–1.15]		[0.63–1.32]	[0.64–1.34]	[0.64–1.34]	[0.59–1.51]
Improved water at age ~8y	1.47		1.59	1.66*	1.70**	1.58
	[0.90–2.40]		[0.98–2.58]	[1.02–2.70]	[1.04–2.77]	[0.85–2.96]
Improved toilets at age ~1y		0.98	1.07	1.08	1.14	1.11
		[0.70–1.36]	[0.78–1.48]	[0.79–1.48]	[0.82–1.57]	[0.75–1.63]
Improved toilets at age ~5y		0.72	0.75	0.76	0.78	0.74
		[0.50–1.05]	[0.52–1.08]	[0.53–1.09]	[0.53–1.14]	[0.47–1.16]
Improved toilets at age ~8y		0.85	0.82	0.85	0.88	0.88
		[0.60–1.21]	[0.58–1.16]	[0.59–1.21]	[0.61–1.26]	[0.57–1.37]

*p < 0 · 05, **p < 0 · 01. All adjusted models include both improved water and improved toilets. Child variables include age in months at outcome and child sex. Household variables are asset index, household size, and household moved between rounds when there is more than one round of data on household toilet and water. Parental variables are age of mother, height of mother, years of schooling of mother, and years of schooling of father. Community variables are urban residence, community population, community wealth, presence of a community hospital, and community has public secondary school

#### Hypothesis 2 (Toilets, stunting, and thinness, unadjusted)

At 1y, 5y, and 8y, children with access to improved toilets were less likely to be stunted than children without access, both concurrently and at subsequent ages (RR = 0.58–0.80; Table 5, column II). 1y olds with access to improved toilets were at reduced risk of thinness at 1y and 5y, relative to 1y olds without access (RR = 0.47–0.49).

#### Hypothesis 3 (Adjustment for covariates)

After adjusting for child-level and household covariates, associations between access to improved water at 1y and less stunting at 5y and 8y remained significant (Table 5, columns III–V). Associations between access to improved toilets and less risk of stunting were attenuated after adjusting for covariates. However, even after adjusting for child, household, parental, and community characteristics, 5y olds with access to improved toilets were significantly more likely to be stunted at 8y. With the exception of access to improved water at 1y and thinness at 8y, there were no significant associations between improved water and thinness once covariates were included. Access to improved toilets at 1y remained significantly associated with reduced thinness at 1y after adjusting for all covariates (RR = 0.40–0.55).

#### Hypothesis 4 (Infancy versus older ages)

Access to improved toilets at 1y was associated with less thinness at 1y and 5y (risk ratios of improved toilets at 1y for thinness at 1y and 5y were 0.55 and 0.53, respectively; Table 5, column VI) but children who had toilet access at 5y and 8y were not significantly more likely than children of the same age with no access to be thin (risk ratios of 0.77 and 0.88, respectively). Thus, inadequate access to improved toilets in infancy was more likely to be associated with less thinness than inadequate toilet access at older ages.

A summary of results for all countries can be found in Table 6. We also examined a pooled model with country interactions on the WASH variables using the full model with all covariates to assess heterogeneity of coefficients across the countries. We found few significant differences in the coefficients, with the following exceptions: for risk of stunting at age 5y, water at age 1y (India ≠ Vietnam), water at age 5y (India ≠ Vietnam), toilets at 1y (India ≠ Vietnam, Ethiopia ≠ Peru, Ethiopia ≠ Vietnam); for risk of stunting at 8y, toilets at 5y (India ≠ Vietnam, Ethiopia ≠ Vietnam); for thinness at age 5y, toilets at 1y (India ≠ Vietnam, Ethiopia ≠ Vietnam); for thinness at 8y, water at 1y (India ≠ Vietnam, Ethiopia ≠ Vietnam), water at 8y (India ≠ Vietnam, Ethiopia ≠ Vietnam).

**Table 6:** Summary of significant associations of stunting (HAZ < −2) and low BMI (zBMI < −2) with household water and sanitation, Young Lives cohort

			Summary			Country							
	Possible Associations	Total Significant Associations	Total Significant Associations, Expected Direction	Total Significant Associations	Total Significant Associations, Expected Direction	Total Significant Associations, Expected Direction (Unadjusted)				Total Significant Associations, Expected Direction (Adjusted)			
		Unadjusted	Unadjusted	Adjusted	Adjusted	Ethiopia	India	Peru	Vietnam	Ethiopia	India	Peru	Vietnam
STUNTING													
Improved water (age ~1y)	12	10	9	1	0	3	0	3	3	0	0	0	0
Improved water (age ~5y)	8	5	5	1	1	0	2	1	2	0	0	0	1
Improved water (age ~8y)	4	2	2	0	0	0	0	1	1	0	0	0	0
Total water (all rounds)	24	16	16	4	4	3	2	5	6	0	0	0	4
Improved toilet (age ~1y)	12	12	12	5	5	3	3	3	3	3	2	0	0
Improved toilet (age ~5y)	8	5	4	1	1	0	0	2	2	0	0	0	1
Improved toilet (age ~8y)	4	2	2	0	0	0	1	0	1	0	0	0	0
Total toilet (all rounds)	24	19	18	6	6	3	4	5	6	3	2	0	1
LOW BMI													
Improved water (age ~1y	12	3	2	2	1	1	0	0	1	0	0	0	1
Improved water (age ~5y)	8	1	1	1	1	1	0	0	0	1	0	0	0
Improved water (age ~8y)	4	0	0	1	0	0	0	0	0	0	0	0	0
Total water (all rounds)	24	5	3	2	1	2	0	0	1	1	0	0	1
Improved toilet (age ~1y)	12	4	4	2	2	1	1	0	2	0	0	0	2
Improved toilet (age ~5y)	8	0	0	0	0	0	0	0	0	0	0	0	0
Improved toilet (age ~8y)	4	0	0	0	0	0	0	0	0	0	0	0	0
Total toilet (all rounds)	24	4	4	2	2	1	1	0	2	0	0	0	2

This table summarizes findings from Tables 2, 3, 4 and 5 where significant refers to the standard *p* < 0.05 level. Total Significant Associations in the Expected Direction refers to associations that are in the direction of what is typically hypothesized (e.g., toilet improvements predict reduced stunting). The number of Possible Associations is the same for unadjusted and (separately) adjusted models.

Additional file 1: Tables S2–S5 present results for the continuous measures of HAZ and BMI, which are consistent with results for the dichotomous measures presented above.

## Discussion

In our analysis of longitudinal data from four low- and middle-income countries we found that children with access to improved water and toilets as their main source were often at reduced stunting risk, compared with age mates without such access. However, access to improved water and toilets was rarely associated with thinness. After adjusting for child, household, parent, and community variables, children without access to improved water were mostly not at greater risk of stunting except in Vietnam where improved water at 1y was associated with less risk of stunting at 1y, 5y, and 8y. In adjusted models, access to improved toilets was significantly associated with fewer stunted children when they were 1y, 5y, and 8y, except for Vietnam. The association between access to improved toilets and lower risk of stunting in infancy was found at age 8y as well. The association between access to improved water and less stunting in infancy sometimes remained for older ages, but associations between access to improved water or toilet and thinness rarely persisted through older ages.

Similar to findings from previous observational studies [16, 28, 29, 31, 35], we found associations between access to improved water and risk of stunting in all but India but these associations were often not significant after adjusting for child, household, parent, and community covariates. Checkley and colleagues [31] found that children with the least access to improved water were the most likely to be stunted, even after adjusting for maternal education and household income. In contrast, Rah and colleagues [35] found that piped water was not associated with reduced odds of stunting. Victora and colleagues [28] report that after accounting for district of residence and income, associations between access to improved water and length-for-age and weight-for-age were largely no longer significant. Our results may underestimate the true associations of improved water with anthropometry because we only measured access, not consumption, and we had no biological indicators of water safety at the point of consumption. Furthermore, our definition of improved water followed the WHO/UNICEF definition that includes a variety of types of water supply ranging from rainwater to piped water. We did not classify as “improved” some sources that may very well have been safe. It may be that there are differing risks associated with different sources of water. Rah and colleagues [35] tested access to piped water, thus maximizing the probability that children categorized as having access to improved water actually were using safe water. They found no association with growth after adjusting for covariates. Even so, in their study, it is possible that some households with no piped water access were actually using safe water. Thus, there was still potential for misclassification of safe water and attenuation of potential effects, so our estimates represent lower bounds.

In unadjusted models, we found numerous associations between improved household toilets and reduced risk of stunting (both concurrently and subsequently), similar to observational studies in Brazil [34], Cambodia [52], Bangladesh [33], India [35], and Peru [31], and in cross-national assessments using DHS data [9, 37]. However, only studies by Lin and colleagues [33], Rah and colleagues [35] and Fink and colleagues [9] present unadjusted and adjusted models, making it possible to ascertain how estimated associations between improved toilets and stunting change with controls for various individual, family, and community factors. In the Lin et al. study [33], greater access to improved toilets was associated with reduced risk of stunting and these associations remained after adjusting for covariates, but were marginally significant. In the Rah et al. study [35], associations between any toilet facility use and the odds of stunting remained significant after adjusting for a range of household, parental and nutritional covariates. In the Fink et al. study [9], children with “high quality” toilet access had significantly and substantially lower odds of being stunted compared to children with “low quality” sanitation, even with adjustments for a range of covariates. A range of factors might influence stunting and thinness, not simply access to improved water and toilets. As noted previously, we adjusted for such household covariates as child’s sex and age; asset index; mother’s height, age, and schooling; father’s schooling; household migration; and urban/rural residence. We also adjusted for community characteristics including presence of a hospital and secondary school, population size, and community wealth. After we adjusted for these child, household, parent, and community variables, improved toilets remained significantly negatively associated with risk of stunting when children were 1y, 5y, and 8y, except in Vietnam. Using a cohort of children, this study is the first to our knowledge to demonstrate the robust and persistent importance of improved toilets in infancy, not only during the first year but continuing into childhood and these results are almost always consistent across four very different countries. It is possible that associations between access to improved toilets (and more broadly, environmental sanitation) and anthropometry may be stronger than what is portrayed here, as we only examine the main source of sanitation for the household; children may be exposed to feces from a variety of sources other than household toilets, including public toilets, shared toilets, other children in the household, inappropriate disposal of water used to clean children’s diapers and bottoms, or chicken and other animal feces.

Our findings correspond with the steady progress seen in key indicators of sanitation and nutrition for the Millennium Development Goals (MDGs) in all four countries over the same time period from 2002 to 2010. The positive correlation seen between increased access to improved water and sanitation and decreased rates of undernutrition in these four countries is reinforced by our findings that show sanitation and nutritional status to be statistically associated, even after controlling for other known factors. Hence, our study gives additional support for the use of such targets for policy makers focused on development.

There may be confounding factors that were not measured in Young Lives. Separately (findings not presented), we examined relations between improved water and sanitation and 1) mother’s completed schooling grades, 2) father’s completed schooling grades, and 3) household consumption. In all four countries, the proportion of children with access to improved water and sanitation increased with mother’s and father’s schooling and consumption.

The strengths of this study include the ability to compare diverse countries, presentation of long-term associations between access to improved water and toilets and child anthropometry, longitudinal data on individual children that permits investigating exposure at early ages on nutritional status at older ages and avoids confounding due to unobserved child or time-varying contextual characteristics of comparing different children across ages in cross-sectional data, and the use of similar household and community measures across ages and countries. Notwithstanding, this study has some limitations. The Young Lives survey data are observational only; therefore, it was not possible to assess impacts of interventions designed to improve water and sanitation. Our study did not include measures of birth length; therefore our associations are not conditional on birth length. Additionally, we were not able to include measures of child hygiene or actual use of improved water and toilets. We also only have information about toilet and water facilities at home but children who go to school or work are also potentially exposed to additional pathogens and we were not able to take this into account. Access to improved water source is used as an indicator of higher probability of safe water, and a recent systematic review and meta-analysis by Bain and colleagues [53] concluded that risk of fecal contamination was significantly lower (OR = 0.15) in improved water sources. However, the authors also found that 38% of the 191 studies they reviewed identified fecal contamination in more than a quarter of the improved water sources that were tested [53]. In a rapid assessment of drinking water quality carried out by the Joint Monitoring Program in Ethiopia, compliance with WHO guideline values and national standards for thermotolerant coliforms and fecal streptococci (72 and 66%, respectively) was inadequate [54]. Thus, the role of improved drinking water in protecting against stunting should be further tested in populations where data on water quality used in households are available.

This study suggests several avenues for future research. These include the need for 1) rigorous longitudinal investigations to determine concurrent and longer-term associations of WASH with stunting and thinness, 2) understanding how average open defecation in a given community (or population sampling unit) is associated with stunting and thinness, 3) examining associations among WASH, children’s schooling, cognition test performance, and other childhood development outcomes, 4) additional information about water quality used in households, 5) a better understanding of how different caregiving practices and physical environments differentially influence child growth, and 6) intervention research to examine how other factors might explain the magnitude of impact of programs designed to promote latrine construction and use. These factors could include inadequate program coverage; insufficient latrine use; the presence of rotavirus and zoonotic agents that are only partly prevented by sanitation; and whether latrines are effective at containing excreta. Additionally, it is possible, using Young Lives or other longitudinal data, to examine changes from one round to the next in individuals’ access to improved water and toilets and how changes in access might be associated with changes in stunting and thinness.

With respect to programs and policies, additional studies are needed before drawing definitive conclusions about the impact of toilets relative to water. Such studies should address potential misclassification of improved water in particular. Research by Smith and Haddad [31] suggests that the intervention mix needed likely varies but that in South Asia and Sub-Saharan Africa, sanitation access should be prioritized because of its strong association with stunting and because sanitation coverage is very low. However, as Patil and colleagues [43] note, it is difficult to achieve sufficiently large improvements in sanitation to produce expected health benefits. This lends encouragement to interventions to improve hygiene and in particular toilets at both household and community levels in order to affect chronic malnutrition and lifelong stunting with all its adverse consequences.

## Conclusions

Our results indicate that access to improved toilets has fairly broad and significant predictive power for less risk of stunting. Despite different cultures and child-rearing practices in four diverse contexts, we found direct and robust associations between access to improved toilets and reduced risk of stunting, concurrently and later.

## Additional files

Additional file 1: Timing of measurement of outcomes, exposures, and other covariates and OLS regression results. (DOC 359 kb)

## Abbreviations

BMI-Z: Body mass index z-score
DHS: Demographic and Health Surveys
EE: Environmental enteropathy
HAZ: Height-for-age z-score
WASH: Water, sanitation, and hygiene
WAZ: Weight-for-age z-score
YL: Young Lives

## Footnotes

1 The United Nations definition of sanitation includes collection, storage, treatment, disposal, reuse, and recycling of human excreta; drainage, disposal, recycling, and re-use of wastewater, stormwater and household, industrial and hazardous solid waste. Young Lives includes household access to improved water and improved toilets. We use ‘improved toilets’ and ‘improved water,’ where ‘improved’ means that children had access to improved water or toilets, *not* that access improved between data collection rounds. We do not know which children were consuming safe water, as improved water may be contaminated between sources and use.

2 Information on handwashing is not available for all four countries so associations between handwashing and children’s nutritional status are not addressed.

3 We constructed asset indices [55] using first principal components of nine consumer durables (bike, car, fridge, mobile phone, motorbike, telephone, radio, sewing machine, television), five housing quality indicators (cooking fuel quality, roofing material, wall material, floor material, electricity availability), household ownership, and number of rooms per person. The first principal component is consistent with 23.7% of the variance for Ethiopia, 22.5% for India, 26.0% for Peru and 26.0% for Vietnam.

4 Community data were reported by community leaders.

5 We constructed “community wealth” as average asset indices for other households in the same cluster.

6 We were not able to allow error clustering in binomial analysis because the modified Poisson regression estimation requires use of the sandwich estimator of variance. We allowed for clustering by community in the continuous analysis of HAZ and BMI (Additional file 1: Tables S2–S5), using the Stata robust command.
