# Supplementary material for: Children with access to improved sanitation but not improved water are at lower risk of stunting compared to children without access: a cohort study in Ethiopia, India, Peru, and Vietnam
Source: BMC Public Health. 2017 Jan 23;17:110. doi: 10.1186/s12889-017-4033-1 (PMC5259877; doi:10.1186/s12889-017-4033-1)
Supplement: Additional file 1: — Timing of measurement of outcomes, exposures, and other covariates and OLS regression results. (DOC 359 kb) [file 12889_2017_4033_MOESM1_ESM.doc]

**Supplementary Tables**

Table S1.Timing of measurement of outcomes, exposures, and other covariates

Table S2. Ordinary least squares regression models of HAZ and zBMI on improved water and sanitation facilities, Young Lives cohort: Ethiopia

Table S3. Ordinary least squares regression models of HAZ and zBMI on improved water and sanitation facilities, Young Lives cohort: India

Table S4. Ordinary least squares regression models of HAZ and zBMI on improved water and sanitation facilities, Young Lives cohort: Peru

Table S5. Ordinary least squares regression models of HAZ and zBMI on improved water and sanitation facilities, Young Lives cohort: Vietnam

**Table S1. Timing of measurement of outcomes, exposures, and other covariates**

|  | **Round 1**  **(~1y old)** | **Round 2**  **(~5y old)** | **Round 3**  **(~8y old)** |
| --- | --- | --- | --- |
| **Outcomes** (child level) |  |  |  |
| Stunting | X | X | X |
| Thinness | X | X | X |
|  |  |  |  |
| **Exposure** (household level) |  |  |  |
| Improved water | X | X | X |
| Improved toilets | X | X | X |
|  |  |  |  |
| **Other covariates** |  |  |  |
| Child’s age in months | X | X | X |
| Child’s sex | X |  |  |
| Average household consumption |  | X | |
| Mother’s height | X |  |  |
| Mother’s completed schooling (grades attained) |  | X |  |
| Father’s completed schooling (grades attained) |  | X |  |
| Mother’s age | X |  |  |
| Urban residence | X |  |  |
| Moved between ages ~1y and ~5y |  | X |  |
| Moved between ages ~5y and ~8y |  |  | X |
| Community wealth | X |  |  |
| Community has hospital | X |  |  |
| Community has secondary school | X |  |  |
| Community population | X |  |  |
|  |  |  |  |

* All community level exposure variables are linked to the community in which the child lived at age ~1y, the time when exposure is likely most critical, even though some variables are collected at age ~5y. However, this link requires the assumption that these exposure variables remain stable from round 1 (child age ~1y) to round 2 (child age ~5y) in a given community.

**Table S2. Ordinary least squares regression models of HAZ and zBMI on household and community measures of improved water and sanitation facilities, Young Lives cohort: Ethiopia**

|  | **Unadjusted** | **Unadjusted** | **Child Adjusted** | **Child and Household Adjusted** | **Child, Household, and Parent Adjusted** | **Child, Household, Parent, and Community Adjusted** |
| --- | --- | --- | --- | --- | --- | --- |
| **n=1,892** | **I** | **II** | **III** | **IV** | **V** | **VI** |
| **HAZ age ~1y** |  |  |  |  |  |  |
| Improved water age ~1y | 0.28 |  | 0.19 | 0.07 | 0.07 | 0.08 |
|  | [-0.11 - 0.67] |  | [-0.20 - 0.59] | [-0.33 - 0.47] | [-0.33 - 0.47] | [-0.30 - 0.46] |
| Improved toilets age ~1y |  | 0.63** | 0.56** | 0.48* | 0.39 | 0.3 |
|  |  | [0.26 - 1.00] | [0.20 - 0.92] | [0.10 - 0.87] | [-0.03 - 0.81] | [-0.06 - 0.65] |
| **HAZ age ~5y** |  |  |  |  |  |  |
| Improved water age ~1y | 0.31* |  | 0.23* | 0.1 | 0.1 | 0.09 |
|  | [0.06 - 0.56] |  | [0.01 - 0.46] | [-0.11 - 0.31] | [-0.10 - 0.30] | [-0.11 - 0.28] |
| Improved water age ~5y | 0.1 |  | 0.06 | -0.02 | -0.01 | 0 |
|  | [-0.08 - 0.28] |  | [-0.12 - 0.24] | [-0.19 - 0.16] | [-0.18 - 0.17] | [-0.14 - 0.14] |
| Improved toilets age ~1y |  | 0.54** | 0.47** | 0.37** | 0.28* | 0.34** |
|  |  | [0.35 - 0.73] | [0.25 - 0.68] | [0.16 - 0.57] | [0.06 - 0.50] | [0.14 - 0.54] |
| Improved toilets age ~5y |  | 0.03 | 0.02 | 0.02 | -0.03 | 0.01 |
|  |  | [-0.12 - 0.19] | [-0.15 - 0.20] | [-0.13 - 0.17] | [-0.16 - 0.11] | [-0.12 - 0.14] |
| **HAZ age ~8y** |  |  |  |  |  |  |
| Improved water age ~1y | 0.26** |  | 0.19* | 0.03 | 0.04 | 0.05 |
|  | [0.09 - 0.44] |  | [0.03 - 0.34] | [-0.12 - 0.17] | [-0.11 - 0.19] | [-0.11 - 0.21] |
| Improved water age ~5y | 0.16* |  | 0.15* | 0.06 | 0.09 | 0.09 |
|  | [0.04 - 0.28] |  | [0.02 - 0.27] | [-0.04 - 0.17] | [-0.02 - 0.19] | [-0.01 - 0.19] |
| Improved water age ~8y | 0.05 |  | 0.06 | -0.03 | -0.05 | -0.04 |
|  | [-0.13 - 0.23] |  | [-0.12 - 0.24] | [-0.20 - 0.13] | [-0.20 - 0.11] | [-0.21 - 0.12] |
| Improved toilets age ~1y |  | 0.42** | 0.34** | 0.21* | 0.16 | 0.19* |
|  |  | [0.27 - 0.56] | [0.19 - 0.49] | [0.04 - 0.38] | [-0.02 - 0.35] | [0.02 - 0.37] |
| Improved toilets age ~5y |  | 0.02 | -0.01 | -0.04 | -0.06 | -0.07 |
|  |  | [-0.11 - 0.15] | [-0.14 - 0.12] | [-0.16 - 0.08] | [-0.18 - 0.06] | [-0.18 - 0.05] |
| Improved toilets age ~8y |  | -0.14 | 1.11 | 0.99 | 1.02 | 1.02 |
|  |  | [-0.33 - 0.06] | [0.91 - 1.35] | [0.81 - 1.21] | [0.84 - 1.25] | [0.83 - 1.27] |
| **zBMI age ~1y** |  |  |  |  |  |  |
| Improved water age ~1y | 0.44 |  | 0.36 | 0.11 | 0.11 | 0.08 |
|  | [-0.05 - 0.92] |  | [-0.10 - 0.81] | [-0.29 - 0.50] | [-0.28 - 0.50] | [-0.23 - 0.40] |
| Improved toilets age ~1y |  | 0.49* | 0.39* | 0.24 | 0.16 | 0.25* |
|  |  | [0.12 - 0.86] | [0.07 - 0.71] | [-0.05 - 0.53] | [-0.15 - 0.47] | [-0.03 - 0.53] |
| **zBMI age ~5y** |  |  |  |  |  |  |
| Improved water age ~1y | 0.00 |  | 0.04 | 0 | 0 | 0.01 |
|  | [-0.27 - 0.27] |  | [-0.23 - 0.30] | [-0.27 - 0.27] | [-0.27 - 0.27] | [-0.26 - 0.28] |
| Improved water age ~5y | 0.24 |  | 0.24 | 0.22 | 0.21 | 0.20* |
|  | [-0.03 - 0.52] |  | [-0.04 - 0.52] | [-0.06 - 0.50] | [-0.07 - 0.49] | [-0.04 - 0.43] |
| Improved toilets age ~1y |  | -0.19 | -0.22 | -0.25 | -0.25* | -0.33** |
|  |  | [-0.46 - 0.09] | [-0.50 - 0.06] | [-0.51 - 0.02] | [-0.52 - 0.02] | [-0.58 - -0.08] |
| Improved toilets age ~5y |  | 0.15 | 0.14 | 0.13 | 0.13 | 0.1 |
|  |  | [-0.09 - 0.40] | [-0.09 - 0.37] | [-0.10 - 0.37] | [-0.10 - 0.37] | [-0.09 - 0.29] |
| **zBMI age ~8y** |  |  |  |  |  |  |
| Improved water age ~1y | 0.18 |  | 0.16 | 0.09 | 0.09 | 0.07 |
|  | [-0.00 - 0.36] |  | [-0.01 - 0.34] | [-0.06 - 0.25] | [-0.07 - 0.24] | [-0.09 - 0.23] |
| Improved water age ~5y | 0.04 |  | 0.02 | -0.02 | -0.02 | -0.02 |
|  | [-0.14 - 0.22] |  | [-0.16 - 0.19] | [-0.19 - 0.15] | [-0.19 - 0.15] | [-0.18 - 0.14] |
| Improved water age ~8y | 0.12 |  | 0.11 | 0.07 | 0.06 | 0.05 |
|  | [-0.06 - 0.30] |  | [-0.07 - 0.29] | [-0.11 - 0.25] | [-0.12 - 0.24] | [-0.13 - 0.23] |
| Improved toilets age ~1y |  | 0.12 | 0.06 | 0 | -0.02 | -0.06 |
|  |  | [-0.10 - 0.33] | [-0.14 - 0.25] | [-0.17 - 0.17] | [-0.19 - 0.15] | [-0.24 - 0.12] |
| Improved toilets age ~5y |  | 0.14 | 0.13 | 0.11 | 0.1 | 0.12 |
|  |  | [-0.03 - 0.32] | [-0.04 - 0.29] | [-0.04 - 0.27] | [-0.06 - 0.26] | [-0.04 - 0.28] |
| Improved toilets age ~8y |  | -0.04 | -0.01 | 0.03 | 0.02 | 0.05 |
|  |  | [-0.20 - 0.11] | [-0.15 - 0.14] | [-0.13 - 0.19] | [-0.13 - 0.18] | [-0.11 - 0.21] |

Notes: *p<0•05, **p<0•01. All adjusted models include both improved water and improved toilets. Standard errors allow for clustering by community. Child variables include age in months at outcome and child sex. Household variables are asset index, household size, and household moved between rounds when there is more than one round of data on household toilet and water. Parental variables are age of mother, height of mother, grades attained of schooling of mother, and grades attained of schooling of father. Community variables are urban residence, community population, community wealth, presence of a community hospital, and community has public secondary school.

**Table S3. Ordinary least squares regression models of HAZ and zBMI on household and community measures of improved water and sanitation facilities, Young Lives cohort:** India

|  | **Unadjusted** | **Unadjusted** | **Child Adjusted** | **Child and Household Adjusted** | **Child, Household, and Parent Adjusted** | **Child, Household, Parent, and Community Adjusted** |
| --- | --- | --- | --- | --- | --- | --- |
| **n=1,919** | **I** | **II** | **III** | **IV** | **V** | **VI** |
| **HAZ age ~1y** |  |  |  |  |  |  |
| Improved water age ~1y | 0.13 |  | 0.15 | 0.12 | 0.13 | 0.08 |
|  | [-0.07 - 0.32] |  | [-0.05 - 0.34] | [-0.07 - 0.31] | [-0.05 - 0.31] | [-0.11 - 0.26] |
| Improved toilets age ~1y |  | 0.36** | 0.36** | -0.12 | -0.17 | 0 |
|  |  | [0.15 - 0.57] | [0.17 - 0.56] | [-0.35 - 0.10] | [-0.39 - 0.05] | [-0.26 - 0.25] |
| **HAZ age ~5y** |  |  |  |  |  |  |
| Improved water age ~1y | -0.17** |  | -0.16* | -0.14* | -0.13* | -0.1 |
|  | [-0.29 - -0.04] |  | [-0.28 - -0.04] | [-0.26 - -0.02] | [-0.25 - -0.02] | [-0.22 - 0.02] |
| Improved water age ~5y | 0.26** |  | 0.15 | 0.08 | 0.05 | 0.03 |
|  | [0.07 - 0.44] |  | [-0.03 - 0.34] | [-0.10 - 0.26] | [-0.12 - 0.23] | [-0.15 - 0.22] |
| Improved toilets age ~1y |  | 0.37** | 0.36** | 0.19** | 0.16* | 0.13 |
|  |  | [0.23 - 0.50] | [0.23 - 0.49] | [0.06 - 0.32] | [0.03 - 0.28] | [-0.03 - 0.29] |
| Improved toilets age ~5y |  | 0.20** | 0.20** | -0.01 | -0.02 | -0.03 |
|  |  | [0.07 - 0.32] | [0.07 - 0.32] | [-0.14 - 0.12] | [-0.15 - 0.11] | [-0.16 - 0.10] |
| **HAZ age ~8y** |  |  |  |  |  |  |
| Improved water age ~1y | -0.16* |  | -0.15* | -0.13 | -0.12 | -0.04 |
|  | [-0.29 - -0.02] |  | [-0.30 - -0.01] | [-0.26 - 0.00] | [-0.25 - 0.01] | [-0.18 - 0.09] |
| Improved water age ~5y | 0.34** |  | 0.20* | 0.13 | 0.1 | 0.11 |
|  | [0.14 - 0.53] |  | [0.02 - 0.39] | [-0.06 - 0.31] | [-0.09 - 0.29] | [-0.08 - 0.30] |
| Improved water age ~8y | 0.17 |  | 0.12 | 0.03 | 0.03 | 0.05 |
|  | [-0.10 - 0.45] |  | [-0.15 - 0.38] | [-0.23 - 0.29] | [-0.21 - 0.27] | [-0.19 - 0.28] |
| Improved toilets age ~1y |  | 0.32** | 0.32** | 0.23** | 0.17* | 0.07 |
|  |  | [0.17 - 0.48] | [0.16 - 0.47] | [0.08 - 0.39] | [0.03 - 0.31] | [-0.10 - 0.23] |
| Improved toilets age ~5y |  | 0.15* | 0.14 | 0.03 | 0 | -0.01 |
|  |  | [0.01 - 0.29] | [-0.00 - 0.29] | [-0.12 - 0.17] | [-0.13 - 0.14] | [-0.14 - 0.13] |
| Improved toilets age ~8y |  | 0.28** | 0.28** | 0.12 | 0.12 | 0.12 |
|  |  | [0.15 - 0.42] | [0.15 - 0.42] | [-0.01 - 0.24] | [-0.00 - 0.24] | [-0.01 - 0.24] |
| **zBMI age ~1y** |  |  |  |  |  |  |
| Improved water age ~1y | -0.29** |  | -0.28** | -0.29** | -0.29*** | -0.20** |
|  | [-0.43 - -0.16] |  | [-0.42 - -0.14] | [-0.43 - -0.15] | [-0.43 - -0.15] | [-0.36 - -0.05] |
| Improved toilets age ~1y |  | 0.28** | 0.27** | 0.11 | 0.12 | -0.02 |
|  |  | [0.14 - 0.42] | [0.14 - 0.41] | [-0.04 - 0.27] | [-0.04 - 0.28] | [-0.22 - 0.17] |
| **zBMI age ~5y** |  |  |  |  |  |  |
| Improved water age ~1y | -0.03 |  | -0.05 | -0.05 | -0.05 | -0.06 |
|  | [-0.16 - 0.09] |  | [-0.18 - 0.07] | [-0.17 - 0.07] | [-0.18 - 0.07] | [-0.20 - 0.09] |
| Improved water age ~5y | -0.09 |  | -0.1 | -0.12 | -0.11 | -0.12 |
|  | [-0.31 - 0.14] |  | [-0.32 - 0.13] | [-0.34 - 0.11] | [-0.33 - 0.12] | [-0.36 - 0.11] |
| Improved toilets age ~1y |  | 0.04 | 0.03 | -0.02 | -0.03 | -0.06 |
|  |  | [-0.12 - 0.20] | [-0.13 - 0.18] | [-0.18 - 0.13] | [-0.19 - 0.12] | [-0.24 - 0.13] |
| Improved toilets age ~5y |  | 0.07 | 0.08 | 0.02 | 0.01 | 0.02 |
|  |  | [-0.08 - 0.21] | [-0.07 - 0.23] | [-0.14 - 0.18] | [-0.15 - 0.17] | [-0.13 - 0.17] |
| **zBMI age ~8y** |  |  |  |  |  |  |
| Improved water age ~1y | -0.01 |  | 0 | 0.01 | 0 | -0.04 |
|  | [-0.16 - 0.14] |  | [-0.15 - 0.14] | [-0.14 - 0.16] | [-0.14 - 0.15] | [-0.19 - 0.11] |
| Improved water age ~5y | -0.09 |  | -0.17 | -0.22 | -0.21* | -0.21* |
|  | [-0.33 - 0.15] |  | [-0.41 - 0.06] | [-0.45 - 0.01] | [-0.43 - 0.02] | [-0.45 - 0.02] |
| Improved water age ~8y | -0.13 |  | -0.17 | -0.22 | -0.2 | -0.2 |
|  | [-0.48 - 0.23] |  | [-0.50 - 0.16] | [-0.54 - 0.11] | [-0.53 - 0.13] | [-0.54 - 0.13] |
| Improved toilets age ~1y |  | 0.24** | 0.24* | 0.19* | 0.16* | 0.07 |
|  |  | [0.06 - 0.42] | [0.06 - 0.42] | [0.02 - 0.37] | [-0.01 - 0.33] | [-0.13 - 0.27] |
| Improved toilets age ~5y |  | -0.01 | 0 | -0.06 | -0.09 | -0.14 |
|  |  | [-0.21 - 0.19] | [-0.20 - 0.20] | [-0.26 - 0.14] | [-0.30 - 0.11] | [-0.35 - 0.08] |
| Improved toilets age ~8y |  | 0.22* | 0.24** | 0.15 | 0.12 | 0.1 |
|  |  | [0.05 - 0.40] | [0.06 - 0.41] | [-0.03 - 0.33] | [-0.06 - 0.30] | [-0.07 - 0.28] |

Notes: *p<0·05, **p<0·01. All adjusted models include both improved water and improved toilets. Standard errors allow for clustering by community. Child variables include age in months at outcome and child sex. Household variables are asset index, household size, and household moved between rounds when there is more than one round of data on household toilet and water. Parental variables are age of mother, height of mother, grades attained of schooling of mother, and grades attained of schooling of father. Community variables are urban residence, community population, community wealth, presence of a community hospital, and community has public secondary school.

**Table S4. Ordinary least squares regression models of HAZ and zBMI on household and community measures of improved water and sanitation facilities, Young Lives cohort: Peru**

|  | **Unadjusted** | **Unadjusted** | **Child Adjusted** | **Child and Household Adjusted** | **Child, Household, and Parent Adjusted** | **Child, Household, Parent, and Community Adjusted** |
| --- | --- | --- | --- | --- | --- | --- |
| **n=1,999** | **I** | **II** | **III** | **IV** | **V** | **VI** |
| **HAZ age ~1y** |  |  |  |  |  |  |
| Improved water age ~1y | 0.26* |  | 0.1 | -0.15 | -0.11 | -0.1 |
|  | [0.04 - 0.47] |  | [-0.10 - 0.30] | [-0.34 - 0.04] | [-0.28 - 0.05] | [-0.27 - 0.07] |
| Improved toilets age ~1y |  | 0.47** | 0.43** | 0.12 | 0.08 | 0.05 |
|  |  | [0.25 - 0.68] | [0.24 - 0.63] | [-0.05 - 0.30] | [-0.07 - 0.23] | [-0.09 - 0.18] |
| **HAZ age ~5y** |  |  |  |  |  |  |
| Improved water age ~1y | 0.19* |  | 0 | -0.14* | -0.12* | -0.12* |
|  | [0.01 - 0.36] |  | [-0.14 - 0.14] | [-0.27 - -0.01] | [-0.23 - -0.01] | [-0.24 - -0.01] |
| Improved water age ~5y | 0.43** |  | 0.26** | 0.05 | 0.02 | 0.01 |
|  | [0.23 - 0.63] |  | [0.09 - 0.44] | [-0.11 - 0.21] | [-0.12 - 0.16] | [-0.13 - 0.15] |
| Improved toilets age ~1y |  | 0.48** | 0.39** | 0.22** | 0.14* | 0.1 |
|  |  | [0.30 - 0.67] | [0.23 - 0.56] | [0.08 - 0.36] | [0.02 - 0.26] | [-0.01 - 0.21] |
| Improved toilets age ~5y |  | 0.35** | 0.27** | 0.09 | 0.1 | 0.11 |
|  |  | [0.18 - 0.52] | [0.12 - 0.42] | [-0.04 - 0.23] | [-0.02 - 0.22] | [-0.01 - 0.22] |
| **HAZ age ~8y** |  |  |  |  |  |  |
| Improved water age ~1y | 0.16 |  | 0 | -0.09 | -0.1 | -0.11 |
|  | [-0.01 - 0.32] |  | [-0.16 - 0.16] | [-0.22 - 0.04] | [-0.22 - 0.03] | [-0.23 - 0.02] |
| Improved water age ~5y | 0.31** |  | 0.23* | 0.07 | 0.02 | 0 |
|  | [0.10 - 0.52] |  | [0.03 - 0.42] | [-0.10 - 0.25] | [-0.14 - 0.18] | [-0.15 - 0.16] |
| Improved water age ~8y | 0.32** |  | 0.27** | 0.01 | 0.07 | 0.1 |
|  | [0.15 - 0.50] |  | [0.09 - 0.46] | [-0.17 - 0.20] | [-0.09 - 0.24] | [-0.07 - 0.26] |
| Improved toilets age ~1y |  | 0.44** | 0.42** | 0.23** | 0.14* | 0.11 |
|  |  | [0.28 - 0.60] | [0.27 - 0.58] | [0.10 - 0.36] | [0.03 - 0.26] | [-0.00 - 0.23] |
| Improved toilets age ~5y |  | 0.34** | 0.27** | 0.11 | 0.11 | 0.12* |
|  |  | [0.19 - 0.50] | [0.12 - 0.43] | [-0.02 - 0.24] | [-0.00 - 0.23] | [0.01 - 0.23] |
| Improved toilets age ~8y |  | 0.05 | -0.02 | -0.04 | -0.03 | -0.01 |
|  |  | [-0.14 - 0.23] | [-0.21 - 0.17] | [-0.21 - 0.13] | [-0.18 - 0.13] | [-0.16 - 0.14] |
| **zBMI age ~1y** |  |  |  |  |  |  |
| Improved water age ~1y | 0.19* |  | 0.09 | -0.02 | -0.02 | -0.03 |
|  | [0.02 - 0.36] |  | [-0.08 - 0.27] | [-0.21 - 0.16] | [-0.21 - 0.16] | [-0.20 - 0.14] |
| Improved toilets age ~1y |  | 0.31** | 0.29** | 0.13 | 0.13 | 0.13* |
|  |  | [0.14 - 0.49] | [0.10 - 0.47] | [-0.03 - 0.29] | [-0.03 - 0.29] | [-0.01 - 0.27] |
| **zBMI age ~5y** |  |  |  |  |  |  |
| Improved water age ~1y | 0.12 |  | 0.07 | 0.03 | 0.02 | 0.02 |
|  | [-0.02 - 0.27] |  | [-0.07 - 0.21] | [-0.11 - 0.18] | [-0.11 - 0.16] | [-0.10 - 0.14] |
| Improved water age ~5y | 0.06 |  | 0.05 | -0.02 | -0.03 | -0.02 |
|  | [-0.10 - 0.23] |  | [-0.12 - 0.21] | [-0.19 - 0.16] | [-0.20 - 0.15] | [-0.19 - 0.15] |
| Improved toilets age ~1y |  | 0.17* | 0.15* | 0.1 | 0.09 | 0.11* |
|  |  | [0.04 - 0.31] | [0.02 - 0.29] | [-0.04 - 0.23] | [-0.04 - 0.23] | [-0.01 - 0.23] |
| Improved toilets age ~5y |  | 0.02 | 0 | -0.05 | -0.06 | -0.05 |
|  |  | [-0.12 - 0.16] | [-0.14 - 0.15] | [-0.20 - 0.09] | [-0.21 - 0.09] | [-0.20 - 0.09] |
| **zBMI age ~8y** |  |  |  |  |  |  |
| Improved water age ~1y | 0.19* |  | 0.07 | 0.01 | 0 | 0 |
|  | [0.03 - 0.35] |  | [-0.10 - 0.23] | [-0.16 - 0.18] | [-0.17 - 0.17] | [-0.16 - 0.15] |
| Improved water age ~5y | 0.1 |  | 0.07 | -0.04 | -0.06 | -0.06 |
|  | [-0.07 - 0.27] |  | [-0.09 - 0.24] | [-0.21 - 0.13] | [-0.23 - 0.11] | [-0.23 - 0.11] |
| Improved water age ~8y | 0.26** |  | 0.23** | 0.06 | 0.08 | 0.1 |
|  | [0.08 - 0.43] |  | [0.06 - 0.41] | [-0.11 - 0.24] | [-0.09 - 0.25] | [-0.07 - 0.27] |
| Improved toilets age ~1y |  | 0.38** | 0.35** | 0.22** | 0.20*** | 0.19*** |
|  |  | [0.22 - 0.54] | [0.18 - 0.52] | [0.10 - 0.35] | [0.07 - 0.32] | [0.07 - 0.31] |
| Improved toilets age ~5y |  | 0.09 | 0.04 | -0.06 | -0.07 | -0.08 |
|  |  | [-0.07 - 0.25] | [-0.10 - 0.19] | [-0.20 - 0.07] | [-0.20 - 0.06] | [-0.22 - 0.05] |
| Improved toilets age ~8y |  | 0 | -0.03 | -0.04 | -0.04 | -0.02 |
|  |  | [-0.21 - 0.21] | [-0.25 - 0.20] | [-0.26 - 0.17] | [-0.25 - 0.18] | [-0.22 - 0.18] |

Notes: *p<0·05, **p<0·01. All adjusted models include both improved water and improved toilets. Standard errors allow for clustering by community. Child variables include age in months at outcome and child sex. Household variables are asset index, household size, and household moved between rounds when there is more than one round of data on household toilet and water. Parental variables are age of mother, height of mother, grades attained of schooling of mother, and grades attained of schooling of father. Community variables are urban residence, community population, community wealth, presence of a community hospital, and community has public secondary school.

**Table S5. Ordinary least squares regression models of HAZ and zBMI on household and community measures of improved water and sanitation facilities, Young Lives cohort:** Vietnam

|  | **Unadjusted** | **Unadjusted** | **Child Adjusted** | **Child and Household Adjusted** | **Child, Household, and Parent Adjusted** | **Child, Household, Parent, and Community Adjusted** |
| --- | --- | --- | --- | --- | --- | --- |
| **n=1,905** | **I** | **II** | **III** | **IV** | **V** | **VI** |
| **HAZ age ~1y** |  |  |  |  |  |  |
| Improved water age ~1y | 0.88** |  | 0.75** | 0.29* | 0.29* | 0.31* |
|  | [0.49 - 1.26] |  | [0.38 - 1.13] | [0.02 - 0.55] | [0.07 - 0.51] | [0.03 - 0.59] |
| Improved toilets age ~1y |  | 0.36* | 0.21 | -0.05 | -0.09 | -0.09 |
|  |  | [0.06 - 0.65] | [-0.02 - 0.44] | [-0.23 - 0.13] | [-0.24 - 0.06] | [-0.23 - 0.04] |
| **HAZ age ~5y** |  |  |  |  |  |  |
| Improved water age ~1y | 0.96** |  | 0.86** | 0.64** | 0.54** | 0.28* |
|  | [0.71 - 1.21] |  | [0.57 - 1.14] | [0.38 - 0.90] | [0.35 - 0.73] | [0.06 - 0.50] |
| Improved water age ~5y | 0.14 |  | 0 | -0.18 | -0.17 | -0.19 |
|  | [-0.25 - 0.52] |  | [-0.36 - 0.36] | [-0.49 - 0.14] | [-0.39 - 0.05] | [-0.39 - 0.01] |
| Improved toilets age ~1y |  | 0.21 | 0.07 | -0.01 | -0.07 | -0.09 |
|  |  | [-0.04 - 0.45] | [-0.10 - 0.25] | [-0.16 - 0.14] | [-0.20 - 0.05] | [-0.20 - 0.03] |
| Improved toilets age ~5y |  | 0.29** | 0.21** | 0.02 | -0.04 | -0.06 |
|  |  | [0.11 - 0.47] | [0.06 - 0.36] | [-0.12 - 0.16] | [-0.16 - 0.08] | [-0.19 - 0.06] |
| **HAZ age ~8y** |  |  |  |  |  |  |
| Improved water age ~1y | 0.83** |  | 0.67** | 0.58** | 0.45** | 0.12 |
|  | [0.60 - 1.06] |  | [0.42 - 0.92] | [0.35 - 0.81] | [0.28 - 0.61] | [-0.11 - 0.34] |
| Improved water age ~5y | 0.2 |  | 0.07 | 0.02 | 0.01 | -0.04 |
|  | [-0.04 - 0.44] |  | [-0.16 - 0.30] | [-0.16 - 0.21] | [-0.13 - 0.15] | [-0.18 - 0.11] |
| Improved water age ~8y | 0.23 |  | 0.13 | -0.16 | -0.1 | -0.1 |
|  | [-0.12 - 0.59] |  | [-0.19 - 0.46] | [-0.35 - 0.03] | [-0.25 - 0.05] | [-0.27 - 0.07] |
| Improved toilets age ~1y |  | 0.18 | 0.09 | 0.08 | 0.02 | 0 |
|  |  | [-0.01 - 0.38] | [-0.07 - 0.24] | [-0.07 - 0.23] | [-0.10 - 0.14] | [-0.12 - 0.12] |
| Improved toilets age ~5y |  | 0.29** | 0.23** | 0.19* | 0.13 | 0.11 |
|  |  | [0.14 - 0.43] | [0.09 - 0.37] | [0.04 - 0.33] | [-0.00 - 0.26] | [-0.03 - 0.25] |
| Improved toilets age ~8y |  | 0.19 | 0.14 | -0.1 | -0.11 | -0.12 |
|  |  | [-0.01 - 0.38] | [-0.02 - 0.30] | [-0.25 - 0.05] | [-0.24 - 0.02] | [-0.25 - 0.00] |
| **zBMI age ~1y** |  |  |  |  |  |  |
| Improved water age ~1y | 0.29** |  | 0.21* | 0.21* | 0.23** | 0.15 |
|  | [0.13 - 0.45] |  | [0.02 - 0.40] | [0.01 - 0.41] | [0.03 - 0.42] | [-0.05 - 0.36] |
| Improved toilets age ~1y |  | 0.18** | 0.14* | 0.15* | 0.11 | 0.1 |
|  |  | [0.05 - 0.31] | [0.01 - 0.27] | [0.01 - 0.29] | [-0.03 - 0.25] | [-0.03 - 0.23] |
| **zBMI age ~5y** |  |  |  |  |  |  |
| Improved water age ~1y | 1.21** |  | 1.03** | 0.95** | 0.93*** | 0.44* |
|  | [0.78 - 1.64] |  | [0.58 - 1.47] | [0.51 - 1.39] | [0.50 - 1.37] | [-0.00 - 0.87] |
| Improved water age ~5y | -0.05 |  | -0.16 | -0.21* | -0.21** | -0.17* |
|  | [-0.29 - 0.18] |  | [-0.36 - 0.04] | [-0.41 - -0.01] | [-0.41 - -0.00] | [-0.36 - 0.02] |
| Improved toilets age ~1y |  | 0.33* | 0.20* | 0.18* | 0.15** | 0.13* |
|  |  | [0.05 - 0.62] | [0.05 - 0.36] | [0.04 - 0.33] | [0.01 - 0.30] | [-0.02 - 0.28] |
| Improved toilets age ~5y |  | 0.22* | 0.18** | 0.12* | 0.1 | 0.06 |
|  |  | [0.05 - 0.38] | [0.06 - 0.30] | [0.01 - 0.23] | [-0.02 - 0.23] | [-0.09 - 0.21] |
| **zBMI age ~8y** |  |  |  |  |  |  |
| Improved water age ~1y | 1.35** |  | 1.20** | 1.12** | 0.99*** | 0.55 |
|  | [0.72 - 1.99] |  | [0.56 - 1.83] | [0.49 - 1.74] | [0.41 - 1.56] | [-0.21 - 1.31] |
| Improved water age ~5y | -0.06 |  | -0.14 | -0.17 | -0.19 | -0.21* |
|  | [-0.31 - 0.20] |  | [-0.41 - 0.12] | [-0.43 - 0.08] | [-0.43 - 0.06] | [-0.46 - 0.04] |
| Improved water age ~8y | -0.17 |  | -0.26* | -0.45** | -0.43*** | -0.38*** |
|  | [-0.39 - 0.05] |  | [-0.48 - -0.05] | [-0.63 - -0.27] | [-0.59 - -0.26] | [-0.58 - -0.18] |
| Improved toilets age ~1y |  | 0.18 | 0.02 | 0.02 | -0.05 | -0.07 |
|  |  | [-0.14 - 0.50] | [-0.17 - 0.21] | [-0.17 - 0.21] | [-0.22 - 0.12] | [-0.25 - 0.11] |
| Improved toilets age ~5y |  | 0.21 | 0.16 | 0.13 | 0.07 | 0.04 |
|  |  | [-0.05 - 0.48] | [-0.07 - 0.39] | [-0.09 - 0.35] | [-0.17 - 0.30] | [-0.19 - 0.28] |
| Improved toilets age ~8y |  | 0.14 | 0.22* | 0.05 | 0.03 | 0 |
|  |  | [-0.06 - 0.35] | [0.01 - 0.42] | [-0.14 - 0.25] | [-0.16 - 0.22] | [-0.21 - 0.21] |

Notes: *p<0·05, **p<0·01. All adjusted models include both improved water and improved toilets. Standard errors allow for clustering by community. Child variables include age in months at outcome and child sex. Household variables are asset index, household size, and household moved between rounds when there is more than one round of data on household toilet and water. Parental variables are age of mother, height of mother, grades attained of schooling of mother, and grades attained of schooling of father. Community variables are urban residence, community population, community wealth, presence of a community hospital, and community has public secondary school.
